# What Role do Androgens Play in Endometrial Cancer?

**DOI:** 10.3390/jpm13020341

**Published:** 2023-02-16

**Authors:** Petra Maček, Nikolaus Molinari, Monika Sobočan, Jure Knez

**Affiliations:** 1 Faculty of Medicine, University of Maribor, Taborska ul 8, 2000 Maribor, Slovenia; 2 Division of Gynecology and Perinatology, University Medical Centre Maribor, Ljubljanska ul 5, 2000 Maribor, Slovenia

**Keywords:** endometrial cancer, androgens, testosterone, androstenedione, dehydroepiandrosterone

## Abstract

The role of estrogens and progesterone in the development and progression of endometrial cancer is well-established, but there are very little data about the role of androgens. There are five different androgens produced in women: dehydroepiandrosterone sulphate (DHEAS), dehydroepiandrosterone (DHEA), androstenedione (A_4_), testosterone (T) and dihydrotestosterone (DHT). The most potent hormones are T and DHT, the latter being mainly produced from T in peripheral tissues, including endometrium. Although they are considered to exert antiproliferative effects in many settings and the expression of their receptors is more often associated with a good prognosis in EC, it is still unknown in which specific settings androgens have carcinogenic or protective effects in EC.

## 1. Introduction

Endometrial cancer is the eighth most common cancer worldwide [1]. It is the most common gynecologic cancer in developed countries and represents nearly 5% of cancer cases in women. Contrary to other cancers, the incidence of endometrial cancer and corresponding mortality rates have increased in recent years and are expected to rise during the next decade [2]. This is mainly due to rising obesity rates and diabetes, population aging and decreased use of combined menopausal hormone therapy [3,4]. Since the 1980s, endometrial cancer is commonly categorized into Type 1, which includes estrogen dependent, low grade endometrioid cancers and Type 2, which includes generally hormone independent, high grade non-endometrioid cancers [5]. The World Health Organization (WHO) classification from 2020 and ESGO/ESTRO/ESP guidelines also take into consideration molecular developments in the subclassification of endometrial carcinoma [6,7], which subdivide them into four overarching categories: no specific molecular profile (NSMP), mismatch repair deficient tumors (MMR-d) and ultra-mutated cancers with polymerase epsilon (POLE) mutations (POLE-mut). These three categories mostly coincide with endometrioid histology. The fourth group includes cancers with low mutation rates but high frequency DNA copy number alterations referred to as p-53-abnormal (p53abn), which coincide largely with serous-like carcinoma [5,7]. 

However, clinical decision making still heavily relies on the prior binary classification into Types 1 and 2 endometrial carcinoma [6]. Type I ECs usually express high levels of estrogen receptor α (ER). Type II ECs are less likely to express ER and they have a poorer prognosis [8]. 

Estrogen has a well-known proliferative effect on the endometrium and is considered to have a crucial role of hormone-dependent carcinogenesis, whilst progesterone acts as an antagonist to estrogen by downregulating ER expression, inhibiting active cell division, and promoting cell differentiation through PR. This results in decreased endometrial cancer rates in patients utilizing hormone replacement therapy with progestins in the formulation [5,9]. ER and PR expression in endometrial cancer was also found to impact disease free (DFS) and overall survival (OS) in endometrial cancer [10]. There have been suggestions that androgen receptor expression in endometrial cancer can also contribute to the prognosis. The current body of knowledge shows that androgens impact endometrial diseases (endometriosis, endometrial cancer and endometrial dysfunction) [11]. Levels of androgens have been found to be abnormal in endometrial cancer, which suggests that androgens are closely related to the occurrence and development of EC [12]; however, the evidence about the role of androgens in endometrial cancer development is conflicting. 

The aim of this narrative review is to discuss the role of androgens in endometrial cancer and their impact in different signaling pathways in carcinogenesis.

## 2. Role of Estrogen and Progesterone in Endometrial Cancer

Estrogen is a steroid hormone that has a crucial role in female reproduction, which is primarily exerted through two main nuclear estrogen receptors (ER). Estrogen receptors are members of the nuclear receptor superfamily and consist of five domains. ER has two isoforms: ERα and Erβ [13]. Estrogens promote cell proliferation in endometrial tissue during the proliferative stage of the menstrual cycle. When estrogens are overproduced, this can lead to excessive cell endometrial proliferation [14]. The absence of progestagen action with estrogen predominance is associated with a higher risk of development of endometrial hyperplasia and cancer. Examples of such risk factors are: estrogen-only post-menopause hormone replacement therapy, obesity and polycystic ovarian syndrome (PCOS) [14]. In post-menopausal women, the endometrium becomes more sensitive to estrogen, and consequently the highest incidence of endometrial cancer occurs during the seventh decade of life [14]. 

Progesterone is a steroid hormone that has a key role in coordinating normal mammalian female reproductive physiology. It is primarily secreted by the corpus luteum, which develops in the ovary after ovulation [15]. Progesterone receptor (PR) is a member of nuclear hormone superfamily of ligand-activated transcription factors. PR has two predominant isoforms: PR-A and PR-B [15]. They are both transcribed from the same gene and have the same DNA binding and ligand binding domains, but often have quite different effects on the transcription in the cell [16]. 

The specific roles of PR-A and PR-B in the etiology of endometrial cancer are still not fully understood; however, PR-A expression is increased in ER-positive endometrial cancer. Progesterone induces the expression of anti-mitogen, insulin-like growth factor binding protein I (IGFBP-I) in endometrial cells, but only when it binds on PR-B. Progesterone and its receptors might also mediate anti-proliferative effects through cell-cycle dependent kinases [17]. 

## 3. Physiology of Androgens in Women

Androgens have a vital role in women’s health. Apart from their significance in conversion into estrogens via aromatization, they also have a role in reproductive and non-reproductive tissues in women [18]. 

There are five different androgens in women: dehydroepiandrosterone sulphate (DHEAS), dehydroepiandrosterone (DHEA), androstenedione (A_4_), testosterone (T) and dihydrotestosterone (DHT). The hormones are listed in a descending way regarding their serum concentration, which is opposite to their biological activity [19]. They are mostly derived from cholesterol, which undergoes a conversion by the luteinizing hormone (LH) in theca cells in ovaries and by the adrenocorticotropic hormone in adrenal zona reticularis [19]. The majority of circulating testosterone is reversibly bound to sex hormone-binding globulin (SHBG) (50–60%) and albumin (40–50%). A minor part (1–2%) of plasma testosterone is free and bioavailable [20]. Plasma DHEAS, which is significantly high as a consequence of its binding to albumin and consequently prolonged half-life, provides a large substrate reservoir for conversion to DHEA, androgens and/or estrogens in peripheral tissues [19,20]. 

**Figure 1:**
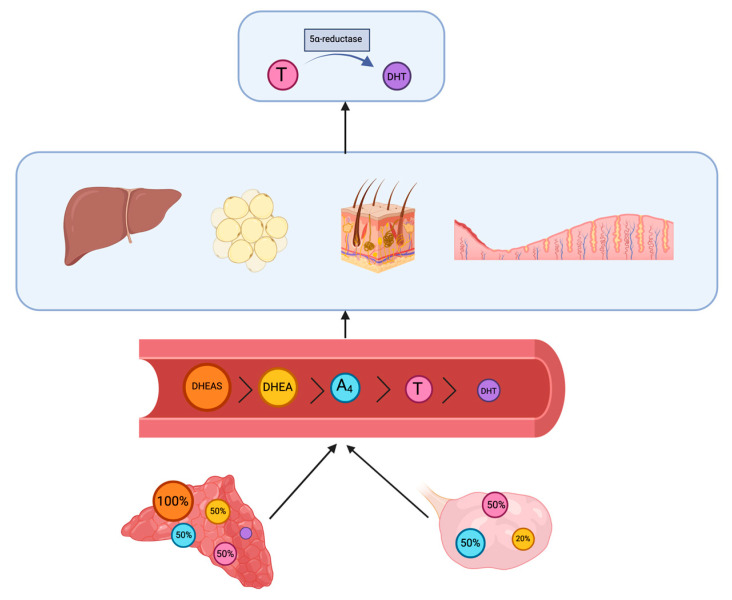
Androgens are produced mostly in the adrenal cortex and the ovaries from cholesterol, apart from DHT, which is mostly synthesized in the peripheral tissues (liver, adipose tissue and the pilosebaceous unit) through conversion from testosterone by 5α-reductase, this conversion also takes place in endometrial tissue After their production, they are released into the blood stream, and their serum concentration ratios are presented in the picture above in a descending order, which is opposite to their biological activity. Created with BioRender.com.

As depicted in Figure 1 the adrenal cortex synthesizes 100% DHEAS, 50% of DHEA, A_4_ and T, and a small amount of DHT. The ovaries produce 50% of A_4_ and T and 20% of DHEA. Additionally, 30% of DHEA comes from conversion from DHEAS in circulation. The greater part of DHT is produced in peripheral tissues, which are the liver, adipose tissue and the pilosebaceous unit, and the transformation is performed by 5α-reductase (5αRD) from testosterone [19]. Additionally, 5α-reductase is also present in endometrial tissue, where this transformation takes place as well [20].

## 4. Androgen Receptors in Endometrial Tissue

Androgen receptors (AR) belong to the nuclear receptor subfamily and to the nuclear receptor superfamily for steroid hormones [21]. They are largely expressed in multiple female tissues (endometrium, ovary, breast, brain, bone and heart) [20]. AR has two isoforms: AR-A and AR-B. Their gene is located on the X-chromosome, and the receptors consist of three domains: transcription regulating domain (N-terminal domain), DNA binding domain, and ligand binding domain (C-terminus domain) [21]. 

It has been postulated that androgens may act on endometrial cell proliferation directly through binding on the AR or indirectly through regulation of insulin growth factor-1 (IGF-1) secretion of the stromal cells, which are located in the endometrial glands [22]. There are similarities in the actions of estrogens and IGF-1, since the latter might also favor endometrial cell proliferation and/or inhibition of apoptosis and therefore increase the risk of EC [22]. Contrary to that, Simitsidellis et al. have demonstrated the antiproliferative effect of androgens on both endometrial epithelial and stromal cells [23,24]. Tuckermann et al. and Brenner et al. investigated the effect of progesterone receptor antagonists on the endometrial epithelium and stroma, and found it upregulates the expression of androgen receptors and resulted in an antiproliferative effect [25,26]. It has been demonstrated that in the normal endometrium, AR is expressed continually throughout the menstrual cycle [22]. 

However, the degree of their expression in the functional layer of endometrium (both in the stroma and epithelial cells) varies with respect to the phase of the cycle [23]. In the proliferative phase, when progesterone levels decrease, the expression of AR increases in epithelial and stromal cells (predominantly in the latter), except the stromal cells of the basal compartment, where it remains unchanged during the cycle [27,28]. On the other hand, AR expression is reduced during the secretory phase, then it is predominantly expressed in epithelium [23,28]. In the postmenopausal (PM) endometrium, the expression of AR differs from premenopausal, since its prevalence is higher in the epithelium and low in the stromal endometrium [29].

Researchers are speculating, based on the results of the studies, that AR might have a cell-specific function in the endometrium. This hypothesis is supported by the antiapoptotic role of stromal AR, and there was no sign of higher cell proliferation in the PM epithelium, although AR expression was upregulated [29]. 

## 5. Androgen Receptors Expression in Endometrial Cancer

Tangen et al. investigated 142 patients with EC, in which they observed the highest level of AR in precursor lesions and well-differentiated primary tumors and a progressive loss of expression in less differentiated tumors. In line with their findings [30], Ito et al. also reported that, with dedifferentiation, there is a loss of AR in the range of 11.4%, as seen in a study of endometrioid primary tumors [30,31], to 79% in a study including primary tumors of different histological subtypes, conducted by Susuki et al. [30,32]. Nonetheless, Hashmi et al., in their study of 89 patients with EC, found no such correlation [33]. Besides progesterone, as mentioned above, androgen also demonstrates antiproliferative effects in normal endometrium. Therefore, it could act as similarly protective to progesterone in estrogen-dependent malignancies, which is demonstrated by poor survival in endometrial carcinoma patients with loss of AR [5,28]. 

Shahin et al., in their retrospective study, where 40 EC Type 1 and 12 EC Type 2 cases were analyzed through AR immunohistochemical expression, and Mahdi et al., in their study with 209 EC Type 1 and 52 EC Type 2 cases analyzed through AR immunohistochemical expression, have come to a similar conclusion that AR expression was more often associated with EC Type I, early tumor stage (I-II), and low FIGO grade (1-2) EC. In addition, Mahdi et al. also found a significant correlation between the AR expression and the absence of lymphovascular invasion and decreased LN involvement, while Shahin et al. did not [22,34].

AR loss was, in the currently available body of knowledge, associated with shorter disease specific survival both in the whole population and within the subgroup of patients with the disease confined to the uterus (FIGO stages I/II) [30]. However, Tanaka et al., in their study of 86 endometrioid endometrial adenocarcinoma (EEA) samples, found that AR status had no independent prognostic value in patients with EEA examined in the study [35].

## 6. Role of Androgens in Endometrial Cancer

Only free androgens are able to diffuse into the cell and exert their biological action by binding to the androgen receptor [36]. 

The research of Michels et al. with 313 patients with endometrial cancer and 354 matched control subjects has shown an increased risk for endometrial cancer to be associated with the highest concentrations of parent androgens—DHEA, androstenedione, and testosterone [37]. However, Clendenen et al., in their study of 161 cases and 303 controls matched on age and date of blood donation, observed no association between androgens and risk of endometrial cancer among women younger than 55 years at the time of diagnosis (and therefore presumed premenopausal or recently menopausal). Nonetheless, they observed a significant increase in endometrial cancer risk among women above 55 years at the time of diagnosis for elevated testosterone and free testosterone concentrations [38]. 

BMI was found to be a strong risk factor for endometrial cancer and is associated with androgens, but the mechanisms of correlation with androgens are not fully understood yet [38]. However, some studies found that there is a positive correlation between the waist-to-hip ratio (WHR) and T, free testosterone (FT) or SHBG concentrations, which are often uncorrelated with the body mass index [39]. The so-called visceral adiposity phenotype can be present in both normal and overweight or obese women. In those women, lower concentrations of SHBG were detected, when compared to their age- and weight-matched counterparts with peripheral or gluteal excess weight or obesity [39]. The different concentrations of SHBG change androgen delivery to target tissues. SHBG levels are regulated by a number of factors. The stimulating factors are estrogens, iodothyronines and growth hormones, while the inhibiting factors are androgens and insulin. Therefore, the higher insulin concentration in visceral adiposity phenotype lowers circulating SHBG, which in turn increases the metabolic clearance rate of SHBG-bound steroids, specifically T, DHT, and androstenedione, the principal active metabolite of DHT. As a consequence, a compensatory elevation of their production rates occurs [39]. Moreover, obesity might also affect the metabolism of androgens, which are not bound to SHBG, since both production and metabolic clearance rates of DHEA and Δ4-androstenedione (Δ4A) are equally increased [39]. 

Insulin resistance (IR) in women might act through several pathways in the development of hyperandrogenism and anovulatory dysfunction. Insulin might have an influence on the pituitary gland to favor the secretion of luteinizing hormone (LH), and it increases adrenal sensitivity to adrenocorticotropin, which in turn increases adrenal androgen production [28,40]. Besides that, insulin and insulin-like growth factor-I receptors possibly act synergistically on theca cells in the ovary to increase the production of androgens [41]. Insulin resistance has two phases: the first compensated and the second uncompensated phase of insulin resistance [42]. In the first phase, hyperinsulinemia leads to a greater production and mitogenic activity of other, insulin-like growth factors, for example IGF-I and IGF-II, which have a significant role in cell proliferation and tumor induction at several sites. In the second phase, hyperglycemia contributes to the increased DNA-synthesis of tumors cells via multiple pathways [42]. All phases of IR are proven risk factors for several cancers, including endometrial cancer, and it also promotes the tumor progression as well. It is possible that the improvement of sexual steroid equilibrium treatment of insulin resistance might be helpful [42]. 

**Figure 2:**
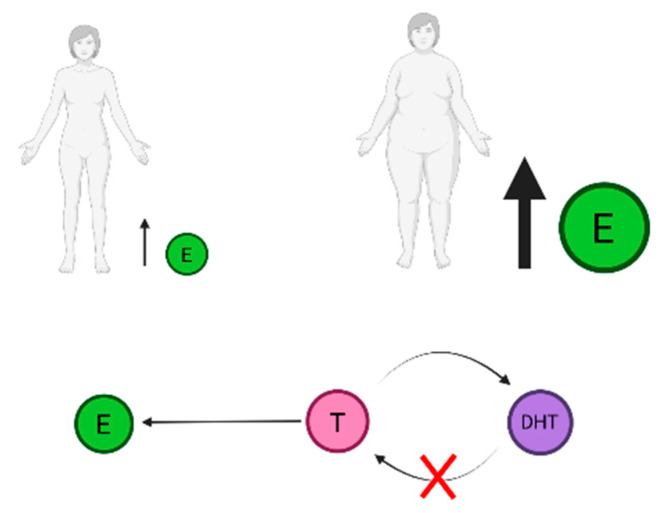
Testosterone can undergo a transformation into estrogen (E) and DHT. Transformation of E from androgens takes place in adipose tissue. Once T is already converted into DHT, the transformation can not be reversed, which leaves less T available for production of E. Increasing DHT relative to its precursor, testosterone, was associated with reduced risk of endometrial cancer, which suggests that higher endometrial cancer risk is associated with the potential for testosterone to be aromatized and that for the etiology of most endometrial cancers, the estrogenic pathway is more important than the androgenic. Created with BioRender.com.

In adipose tissue, aromatization of androgens into estrogens also takes place, which could consequently have an effect on endometrial cancer [38]. Michels et al. observed that the increased risk of endometrial cancer was related to higher parent estrogen concentrations relative to their androgenic precursors [37]. Moreover, because once the DHT is metabolized by 5α-reductase, it cannot undergo aromatization into estradiol or conversion back into testosterone, as depicted in Figure 2, they observed that increasing DHT relative to its precursor, testosterone, was associated with a reduced risk of endometrial cancer [37]. This supports the idea that higher endometrial cancer risk is associated with the potential for testosterone to be aromatized. Finally, all this suggests that, for the etiology of most endometrial cancers, the estrogenic pathway is more important than the androgenic [37]. It is generally accepted that estrogen is the main internal factor that causes EC I, since it mediates endometrial cell growth, proliferation, apoptosis inhibition and angiogenesis through activation of estrogen receptors and down streaming signaling pathways [36]. 

According to Teng et al. in their study of 510 patients with Type I EC and 510 control patients, androgens, and especially testosterone, affect the occurrence of endometrial cancer Type I not only through estrogenic pathways, but also as an independent risk factor and through androgenic pathways, specifically testosterone [36]. It is known that only a free androgen fraction can diffuse into the cell to bind to the AR, where it exerts a carcinogenetic effect [36]. 

## 7. Discussion

The effect of androgens in endometrium is not fully understood, yet as it is seen from multiple contradictory studies.

Some believe androgens have antiproliferative effects on the endometrium, which could be through androgens antagonizing the effects of estrogens [24] or possibly through upregulation of progesterone receptor expression [43]. Nevertheless, other studies concluded that they have proliferative effects, which could be a consequence of upregulation of the epidermal growth factor receptors via androgens in the stromal compartment. Consequently, the increase in the proliferation rate might occur via an indirect effect of the growth factor on stromal cells [44]. 

In our opinion, these inconsistencies could be due to the limited number of participants in the studies or it is possible that androgen receptors have a cell-specific function in the endometrium and therefore have both of those roles, suggesting that there are different types of androgen receptors with different functions. Further investigation with larger study groups is needed.

In line with some other studies, Babayev et al. concluded that androgens upregulate progesterone receptors in the glandular epithelium, whereas they do not increase their expression in human endometrial stromal cells [43]. Therefore, androgens could have an indirect antiproliferative effect on endometrium through upregulation of progesterone receptors.

In a study of breast cancer cells, they investigated the effect of DHT on progesterone receptors [45]. Progesterone receptor A (PR-A) and progesterone receptor B (PR-B) have antagonistic effects in endometrium, since PR-A modulates the anti-proliferative effects of progesterone in the uterus and PR-B in the absence of PR-A leads to cell growth [46]. In the mentioned study, they came to the conclusion that DHT had some transcriptional activity through PR-B, but not through PR-A [45]. This could possibly indicate that a similar role of DHT could also take place in endometrial cells, which could be a factor in endometrial cancer, since we would have to lower DHT concentrations and therefore prevent its proliferative effect through PR-B on endometrium.

Human aromatase is highly specific and the only human enzyme that is capable of catalyzation of aromatization from androgens into estrogens. It catalyzes the conversion of androstenedione, testosterone and 16α-hydroxytestosterone to estrone, estradiol and 17β,16α-estriol, respectively [47]. Studies have shown that, in the etiology of endometrial cancer, the estrogen pathway is more important than the androgen pathway [37], therefore it would be important to prevent the aromatization of androgens into estrogens. 

In future research, it would be important to look into the antiproliferative or proliferative effects of androgens on EC and which of those is more important in its etiology, and whether the influence of one of them might in fact be beneficial in treatment. It would also be important to investigate whether different types of EC react differently to androgens, since we found no such existing studies, and this could possibly be a reason for the contradictory results.

Based on a search on current clinical trials (Table 1), using the keyword endometrial cancer in the category condition or disease and the keyword androgens in the category other terms, we found five ongoing clinical trials, two of them with the status: active, not recruiting, one with the status: not yet recruiting, and two with the status: recruiting, but we found no completed clinical trials.

The first study in Table 1 is researching the AR gene polymorphism and particularly the number of CAG repeats on exon 1 in patients with known endometrial pathologies (benign and malignant). Specific lengths of CAG repeats are associated with greater risk for other testosterone-dependent cancers, such as prostate and breast cancer. This clinical trial aims to correlate the number of CAG repeats on AR with specific endometrial benign or malignant lesions. Other ongoing clinical trials are examining uses of a combination of megestrol acetate with or without metformin. Megestrol acetate blocks estrogen and suppresses the effects of estrogen and androgens. The research explores the effects of drugs decreasing the growth of endometrial intraepithelial neoplasia. The outcome of changed endometrial cell proliferation will be measured by the percentage of Ki-67 positive cells. One of the ongoing clinical trials is also the effect of Gonadotropin-releasing Hormone Agonist (GnRHa) with Letrozole and Diane-35 with Metformin. Through the action of GnRHa, the concentration of testosterone is decreased. Diane-35 combines ethinylestradiol and cyproterone. Ethinylestradiol increases the synthesis of SHBG and therefore decreases the concentration of bioavailable androgens, while cyproterone is a competitive antagonist of AR. These trials will further enhance our understanding of the therapeutic use of androgen receptors in endometrial cancer and potentially represent a future venue for the clinical management of EC.

## 8. Conclusions

From reviewing the existing literature, we conclude that there is still much that is unknown about the effect of androgens on the endometrium, and more studies should be conducted, so we can effectively treat endometrial cancer. Androgens, most importantly testosterone and DHT, and their receptors could be possible targets for future hormone treatments. T can be transformed into DHT or E, and the later contributes to the estrogenic pathway, which is an important factor in the etiology of endometrial cancer. Transformation of T into DHT, by 5α-reductase, is irreversible, which leaves less T available for transformation into E. We should furthermore explore the effect DHT has on endometrial tissue. Depending on the results, in case that DHT does not contribute to the development of endometrial cancer, we should possibly consider whether it would be beneficial to enhance the transformation of T into DHT, since it would reduce the effect of the endometrial pathway, but the effects of the subsequent rise in DHT on patients and their side effects should also be considered.

## Figures and Tables

**Table 1:** Ongoing clinical trials * involving androgens in endometrial cancer.

Target	Therapeutic Agent	Carcinoma	Trial Drug Design	Reg. Nr.
CAG repeats on exon 1	DNA analysis	Benign endometrial pathology	Benign lesions of the endometrium vs. control group	NCT05157373
		Malignant endometrial pathology	EC vs. control group	NCT05157373
AR	enzalutamide	eEC stage III-IV or recurrent cancer	Experimental: Treatment (enzalutamide, paclitaxel, carboplatin)	NCT02684227
Ki-67 positive cells	megestrol acetate, metformin	endometrial intraepithelial neoplasia	megestrol acetate with or without metformin	NCT04576104
aromatase	exemestane	complex EAH/ endometrial intraepithelial neoplasia	Single experimental arm: exemestane treatment	NCT03300557
		low grade EC		
progestin-insensitive cells	GnRHa + letrozole	progestin-insensitive EEC and EAH	Patients with EEC or EAH	NCT05316935
	Diane-35 + metformin	progestin-insensitive EEC and EAH	Patients with EEC or EAH	NCT05316935

* Accurate data as of 12 February 2023. Abbreviations: EC: endometrial cancer, AR: androgen receptor, eEC: Endometrioid Endometrial Cancer, EEC: early stage endometrial cancer, EAH: atypical hyperplasia, GnRHa: Gonadotropin-releasing Hormone Agonist.

## Data Availability

Not applicable.
